# *Zanthoxylum bungeanum* Seed Oil Attenuates LPS-Induced BEAS-2B Cell Activation and Inflammation by Inhibiting the TLR4/MyD88/NF-*κ*B Signaling Pathway

**DOI:** 10.1155/2021/2073296

**Published:** 2021-09-24

**Authors:** Jing Hou, Jun Wang, Jingyi Meng, Xiaoting Zhang, Yuanjing Niu, Jianping Gao, Yun'e Bai, Jiangtao Zhou

**Affiliations:** School of Pharmaceutical Science, Shanxi Medical University, Taiyuan, Shanxi Province, China

## Abstract

**Background:**

*Zanthoxylum bungeanum* seed oil (ZBSO) is a natural essential oil derived from the seeds of the Chinese medicinal plant *Zanthoxylum bungeanum*, which has been investigated for antitumor and anti-inflammatory effects. However, little is known regarding the effects of ZBSO in chronic obstructive pulmonary disease (COPD).

**Methods:**

In this study, lung epithelial cells (BEAS-2B) were induced by lipopolysaccharide (LPS) to establish an *in vitro* model of COPD, and cytotoxicity was detected by a cell counting kit 8 (CCK-8) assay. Griess test, enzyme-linked immunosorbent assay (ELISA), reverse transcriptase quantitative polymerase chain reaction (RT-qPCR), western blot, immunofluorescence, and molecular docking analyses were used to investigate the effects of ZBSO and its potential mechanisms.

**Results:**

The results showed that LPS promoted the expression of nitric oxide (NO), reactive oxygen species (ROS), malondialdehyde (MDA), tumor necrosis factor-*α* (TNF-*α*), interleukin-6 (IL-6), monocyte chemoattractant protein-1 (MCP-1), matrix metalloproteinase-2 (MMP-2), MMP-9, cyclooxygenase-2 (COX-2), and prostaglandin E_2_ (PGE2), suggesting that LPS can induce inflammation and oxidative stress in BEAS-2B cells. ZBSO inhibits the LPS-induced expression of inflammatory mediators and proinflammatory cytokines in BEAS-2B cells. The molecular docking results indicated that active components in ZBSO could successfully dock with toll-like receptor 4 (TLR4), myeloid differentiation factor 88 (MyD88), and p65. Immunofluorescence and western blot analyses further demonstrated that ZBSO repressed protein expression associated with the TLR4/MyD88/nuclear factor-*κ*B (NF-*κ*B) signaling pathway.

**Conclusions:**

ZBSO reduced the inflammatory response and oxidative stress induced by LPS by inhibiting the TLR4/MyD88/NF-*κ*B signaling pathway, thereby suppressing COPD. ZBSO may represent a promising therapeutic candidate for COPD treatment.

## 1. Introduction

According to data from the Global Initiative for Chronic Obstructive Lung Disease (GOLD), chronic obstructive pulmonary disease (COPD) is a common, preventable, and treatable respiratory disease [1]. Because of the aging of the population, increased air pollution, smoking, and other factors, the incidence of COPD has increased, becoming the fourth leading cause of death worldwide [2]. COPD symptoms include airflow obstruction and difficulty breathing, including emphysema, chronic bronchitis, and asthma [3]. The primary cause of COPD development is tobacco smoke, but factors including environmental exposure and genetic risk may worsen the COPD course [4]. Airway inflammation is the primary pathological feature of COPD and has been implicated in the pathogenesis and progression of COPD; therefore, anti-inflammatory therapy is the typical treatment strategy for COPD [5]. Glucocorticoids, which have significant anti-inflammatory effects, have been increasingly used to treat COPD in recent years [6]. However, glucocorticoids are associated with numerous and serious side effects, warranting a search for safe alternative therapeutic agents [7].

Inflammation is the initial response of the body's innate immune system to external infections and stimuli. Pathogens, chemical immune responses, damaged cells, irritants, etc. can all induce inflammation. However, excessive inflammation can lead to disorders of the body, excessive production of oxygen free radicals, and ultimately destruction of homeostasis, which often leads to diseases such as fever, COPD, arthritis, and even cancer [8]. The toll-like receptor 4 (TLR4)/myeloid differentiation factor 88 (MyD88)/nuclear factor-*κ*B (NF-*κ*B) signaling pathway is involved in the development of the COPD airway inflammation [9]. Lipopolysaccharide (LPS) is an outer cell wall component found in Gram-negative bacteria that is well known to induce an inflammatory process mediated by the transmembrane receptor TLR4 [10]. When TLR4 recognizes LPS, a cascade of reactions occurs, including the TLR4-mediated activation of downstream NF-*κ*B signaling pathway via MyD88 [11, 12]. NF-*κ*B is a key regulator of inflammatory dysregulation and is necessary for the transcription of inflammatory cytokines, such as interleukin (IL)-6, IL-1*β*, and tumor necrosis factor-*α* (TNF-*α*) [13]. The activation of NF-*κ*B by TLR4 is thought to play a key role in COPD pathogenesis [14].

Medicinal plants have been used for thousands of years in Asia and have traditionally played an important role in primary health care. A large number of plant extracts and bioactive compounds derived from medicinal plants have been shown to be effective in the treatment of COPD [15, 16], such as betulin [17] and Liu-Jun-Zi-Tang [18]. Therefore, effective drugs for COPD treatment may be derived from traditional Chinese medicine. *Zanthoxylum bungeanum* is a common food additive and herbal medicine used in China and is widely distributed across Asian countries [19]. In 2002, the Chinese Ministry of Health officially approved *Zanthoxylum bungeanum* Maxim. (ZBM) as a dietary, medicinal herb for improved public health [20]. *Zanthoxylum bungeanum* seeds, a byproduct of *Zanthoxylum bungeanum*, are produced at a rate of approximately 1 million tons each year and are often viewed as a byproduct or waste fuel. To date, studies have revealed the pharmacodynamic properties of *Zanthoxylum bungeanum* seed oil (ZBSO) in inflammatory diseases, such as asthma and burn [21, 22] and antitumor effects [20, 23, 24]. However, the effects of ZBSO and mechanisms through which ZBSO acts on airway inflammation, a common symptom of COPD, remain unclear.

Based on the relationship between COPD pathogenesis and the TLR4 signaling pathway, the anti-COPD effects of ZBSO may be associated with the inhibition of TLR4/MyD88/NF-*κ*B signaling pathway activation and the prevention of excessive oxidative stress generation. This paper will provide a basis for further understanding the potential mechanism through which ZBSO mediates the treatment of COPD.

## 2. Materials and Methods

### 2.1. Reagents and Chemicals

LPS was obtained from Solarbio (055: B5, Beijing, China). The antibodies against MyD88 (23230-1-AP), NF-*κ*B p65 (10745-1-AP), and phospho-NF-*κ*B p65 were obtained from Proteintech (Hubei, China). The antibodies against TRL4 (GB11519) and *β*-actin were gained from Servicebio (Hubei, China). All secondary antibodies used for western blot were purchased from ImmunoWay (Plano, TX). Cy3-conjugated goat anti-rabbit IgG (H + L) used in immunofluorescence experiments was obtained from Servicebio (Hubei, China).

### 2.2. Preparation of ZBSO

*Zanthoxylum bungeanum* seeds were purchased from Yuncheng City, Shanxi Province, and identified as *ZBM* by Professor Yun'e Bai (School of Pharmaceutical Science, Shanxi Medical University) in August 2018. The samples were stored in the Herbarium of Traditional Chinese Medicine, School of Pharmacy, Shanxi Medical University. The extraction and separation methods used to obtain ZBSO were based on previous research performed in our laboratory [25, 26]. In short, the *Zanthoxylum bungeanum* seeds (100 g) were pulverized and extracted twice, using 800 mL and 600 mL 95% ethanol (Sanwei, Henan, China). The filtrates were combined and concentrated under reduced pressure, and the ZBSO content was calculated. After filtering through a 0.22 *μ*m filter membrane, the extract was stored in the dark at −20°C. This extract was used in all experiments described in this study. The final concentration of dimethyl sulfoxide (DMSO) does not exceed 0.05%.

### 2.3. Cell Culture and Treatment

The human lung epithelial cell line BEAS-2B (EK-Bioscience, Shanghai) was maintained in Dulbecco's modified Eagle's medium (DMEM) supplemented with 10% fetal bovine serum (FBS) and 1% penicillin/streptomycin in a 95% air and 5% CO_2_ environment at 37°C. In all experiments, ZBSO (0.025%, 0.05%, and 0.1%) or indomethacin (the positive control drug) were administered prophylactically for 4 h, followed by continued exposure to 100 ng/mL LPS for 24 h.

### 2.4. Cell Viability Assay

ZBSO (0.025%, 0.05%, and 0.1% *v/v*) was applied to cells plated in a 96-well plate at a cell density of 5 × 10^4^ cells/well. After 24 h, CCK-8 was added to each well and incubated for 2 h at 37°C. Absorbance (optical density (OD) values) was measured at 450 nm.

### 2.5. Griess Reagent Assay

BEAS-2B cells were cultured overnight in 96-well plates at a density of 5 × 10^4^ cells/well. The cells were treated with indomethacin (5 *μ*M) or ZBSO (0.025%, 0.05%, and 0.1% *v/v*) for 4 h, followed by incubation with 100 ng/mL LPS for 24 h. The supernatant was collected, and nitrite levels were measured using the Griess method, according to the manufacturer's instructions (Elabscience, Wuhan, China).

### 2.6. Dichlorodihydrofluorescein Diacetate (DCFH-DA) Assay

BEAS-2B cells (10^6^ cells per 1 mL medium) were treated for 4 h with ZBSO (0.025%, 0.05%, and 0.1% *v/v*), followed by incubation with or without 1 *μ*g/mL LPS for 24 h. Dichlorodihydrofluorescein diacetate (DCFH-DA) staining was used to measure total intracellular reactive oxygen species (ROS) levels according to the manufacturer's protocol (Elabscience, Wuhan, China). In brief, the cell culture medium was removed, DCFH-DA was added at a final concentration of 10 *μ*M, and the cells were incubated at 37°C in the dark for 1 h. Cells were washed with phosphate-buffered saline (PBS) 3 times to completely remove excess DCFH-DA, and ROS production was observed by inverted fluorescence microscopy (magnification × 20; Leica, Germany) and quantified using ImageJ software version 1.46 (National Institutes of Health, Bethesda, MD, USA). The experiments were repeated in triplicate.

### 2.7. Analysis of Antioxidative Enzymatic Activities

BEAS-2B cells were stimulated with ZBSO (0, 0.025%, 0.05%, and 0.1% *v/v*) for 4 h. The positive control group (indomethacin 5 *μ*M) and ZBSO-treated groups were then exposed to LPS (100 ng/mL) for 24 h. The cellular activities of superoxide dismutase (SOD), glutathione peroxidase (GSH), and malondialdehyde (MDA) were determined using a commercial kit, according to the manufacturer's instructions (Elabscience, Wuhan, China).

### 2.8. Real-Time Polymerase Chain Reaction (RT-PCR)

The total RNA in BEAS-2B cells was extracted using a TransGen (Beijing, China) TransZol Kit and quantified using Eppendorf Bioluminometer D30. A total of 1 *μ*g RNA was used to synthesize the corresponding cDNA using a one-step gDNA Removal and cDNA Synthesis SuperMix Kit (TransGen, Beijing, China). SYBR Green-based real-time PCR experiments were performed to detect the total mRNA transcripts on a LightCycler 96 Real-Time PCR System (Roche, Mannheim, Germany) platform.  Table 1 shows the primer sequences. Gene expression was calculated as previously described, using the 2^−ΔΔCt^ method.

### 2.9. ELISA Assay for COX-2 and PGE2

The culture medium was collected, and the levels of cyclooxygenase 2 (COX-2) and prostaglandin E_2_ (PGE2) were determined under different conditions using an ELISA, according to the manufacturer's protocol. The absorbance at 450 nm and 570 nm were measured with a microplate analyzer (SpectraMax^®^190, USA). The absolute value was obtained using a standardized 4-parameter logistic curve.

### 2.10. Molecular Docking

The oral bioavailability (OB) of ZBSO was evaluated by the OBioavail 1.1 model in the traditional Chinese medicine systems pharmacology (TCMSP) database (http://ibts.hkbu.edu.hk/LSPtcmsp.php). Drug-likeness (DL) was analyzed using a model from the TCMSP database, which was constructed according to the molecular descriptors and Tanimoto coefficient [25]. Active ingredients were screened using OB ≥ 30% and DL ≥ 0.18 as cutoff parameters [27]. The Tripos Mol2 type files for selected active ingredients were searched using the TCMSP database. Active ingredients were imported into Discovery Studio 3.5 software for hydrogenation optimization and saved in .pdb format as a ligand for backup. The three-dimensional structure of the target protein (Table 2) was obtained from the Protein Database (PDB) (https://www.rcsb.org/pdb/home/home.do), and AutoDock Tools software was used to remove water, hydrogenate the protein, add atomic charges, and set the atom type, which was then saved in .pdbqt format for use as a recipient. AutoDock 1.5.6 software was used to conduct molecular docking between the compounds and protein receptors, and the docking results were analyzed. Binding energy≤−5.0 kJ/mol was used as the screening criteria.

### 2.11. NF-*κ*B P65 Immunofluorescence Assay

The cells were pretreated with ZBSO (0.025%, 0.05%, and 0.1% *v/v*) and positive control for 4 h and then stimulated with LPS (100 ng/mL) for 24 h. Untreated cells served as a control. Subsequently, the cells were immobilized in 4% paraformaldehyde (Beyotime, Beijing) and permeated in PBS containing 0.1% Triton X-100. The cells were sealed with 5% goat serum for 1 h at room temperature, followed by incubation with primary anti-NF-*κ*B p65 antibody at 4°C overnight. Cells were washed 3 times and labeled with Cy3 at room temperature for 1 h. Finally, Hoechst 33258 (1 *μ*g/mL) was added and incubated in the dark at 37°C for 30 min. Images were taken using a fluorescence microscope (Olympus X81, Tokyo, Japan) with excitation/emission wavelengths of 490 nm/540 nm for Cy3 and 360 nm/450 nm for Hoechst 33258. All morphometric measurements were determined by at least three independent individuals in a blinded manner.

### 2.12. Western Blot Analyses

The collected BEAS-2B cells were washed 3 times with precooled PBS, followed by the addition of 150 *μ*L radioimmunoprecipitation assay (RIPA) lysis buffer (lysis buffer: phenylmethylsulfonyl fluoride [PMSF]: protein phosphatase inhibitor = 100 : 1 : 1). After incubation in an ultrasonic cell crusher ice bath for 6 min, samples were centrifuged (12000 × g) at 4°C for 10 min to collect the supernatant. The extracted proteins were quantified by using a bicinchoninic acid (BCA) protein assay kit (Thermo, Waltham, MA, USA). The protein concentrations were adjusted using lysis buffer. Protein loading buffer was added (total protein: loading buffer = 4 : 1) and heated for 5 min at 100°C, and then the samples were cooled to room temperature and stored at −20°C. Equal amounts of sample protein were separated by electrophoresis (8% SDS-PAGE) and transferred to polyvinylidene difluoride (PVDF) membranes. The membranes were blocked with 5% milk for 1 h at room temperature and incubated with primary antibodies against TLR4 (1 : 1000), MyD88 (1 : 4000), p65 (1 : 3000), p-p65 (1 : 2000), histone 3 (1 : 1000) and *β*-actin (1 : 2000) at 4°C overnight. After washing adequately with Tris-buffered saline containing Tween 20 (TBST) three times, the membranes were incubated with secondary antibodies (Goat anti-Rabbit IgG-HRP) at room temperature for 1 h. The membranes were washed three times and exposed to a gel imager using an enhanced chemiluminescence (ECL) kit. The bands were visualized with a Bio-Rad ChemiDoc XRS. The density of the western blot bands was quantified using Quantity One software (Bio-Rad, CA, USA).

### 2.13. Statistical Analysis

The data are expressed as the mean ± standard deviation (SD) of three independent experiments. Statistical analyses were performed using one-way analysis of variance followed by a least significant difference (LSD) test for multiple comparisons using SPSS 26.0 software (IBM, New York, USA) and GraphPad Prism 8.3 (GraphPad Software, Inc., La Jolla, CA, USA). Student's *t*-test was used to evaluate comparisons between two groups. A *p* value of <0.05 was considered significant.

## 3. Results

### 3.1. ZBSO Exhibits Little Cytotoxicity in BEAS-2B Cells

**Figure 1:**
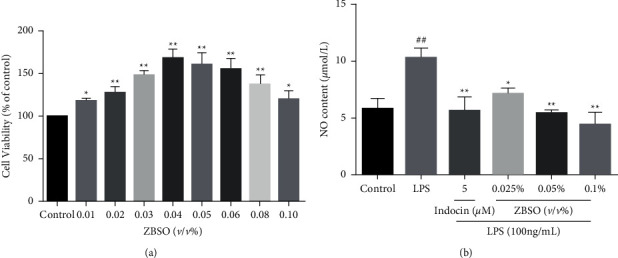
BEAS-2B cells were treated with different concentrations of ZBSO (0.01%–0.1% *v/v*) for 24 hours. CCK-8 assay was used to detect cell viability. (a) The level of NO in the medium was determined by the Griess method. (b) Each point represents the mean ± SD of three experiments; ^#^*p* < 0.05 and ^##^*p* < 0.01 versus the control group; ^*∗*^*p* < 0.05 and ^*∗∗*^*p* < 0.01 versus the LPS group.

The extraction method used to obtain ZBSO was based on previous research. The oil yield for ZBSO was 23.2%, which was equivalent to 0.8898 g of crude drug per mL, and the main components were unsaturated fatty acids (19.82%–28.08%) [28]. To investigate the cytotoxicity of ZBSO in human lung epithelial cells, BEAS-2B cells were treated with various doses of ZBSO, ranging from 0.01% to 0.1%, for 24 h. The results, shown in Figure 1(a), revealed that ZBSO concentrations below 0.1% have no inhibitory effects on BEAS-2B cell growth. Therefore, to avoid affecting cell growth and ensure the effectiveness of the drug, the final tested concentrations used for ZBSO in the present study were 0.025%, 0.05%, and 0.1% *v/v*. The effects of low, medium, and high ZBSO doses were examined on LPS-induced changes in nitrite levels in BEAS-2B cells, which revealed that ZBSO could significantly inhibit NO generation (Figure 1(b)), inhibiting the inflammatory response.

### 3.2. ZBSO Protects BEAS-2B Cells against LPS-Induced Oxidative Stress

**Figure 2:**
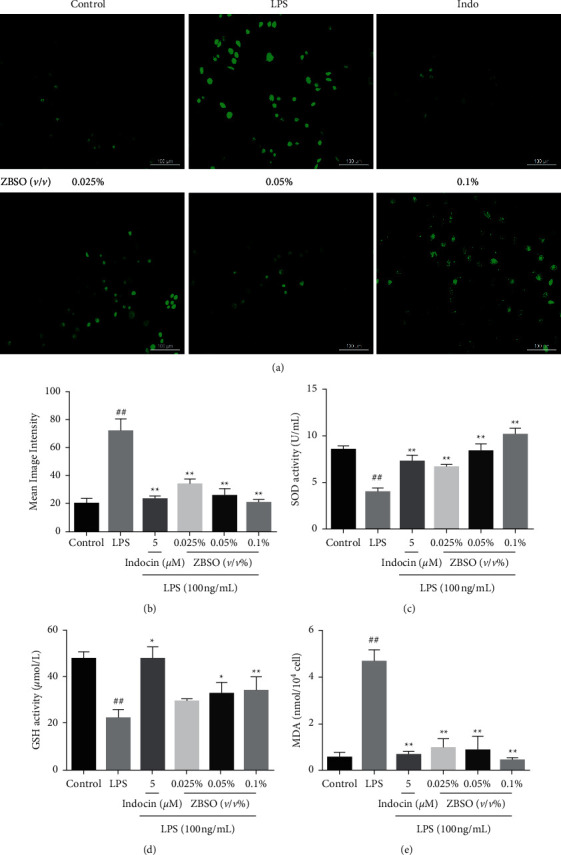
ZBSO protects BEAS-2B cells from LPS-induced oxidative stress. DCFH-DA staining (a, b) was used to detect intracellular reactive oxygen species (ROS), and the levels of the antioxidants SOD (c), GSH (d), and MDA (e) were detected using a commercial kit. The experiment was repeated three times. The results are expressed as the mean ± SD; ^#^*p* < 0.05 and ^##^*p* < 0.01 versus the control group; ^*∗*^*p* < 0.05 and ^*∗∗*^*p* < 0.01 versus the LPS group.

To elucidate the effects of ZBSO on LPS-induced oxidative stress in BEAS-2B cells, we examined the intracellular levels of ROS, SOD, GSH, and MDA. As shown in Figure 2, the levels of ROS and MDA in the LPS group significantly increased compared with those of the control group (*p* < 0.05). However, compared with the LPS-treated group, ZBSO and indomethacin treatment significantly alleviated oxidative stress, as indicated by reduced intracellular ROS and MDA levels (*p* < 0.01 − 0.05). The levels of SOD and GSH in the LPS group significantly decreased compared with those in the control group (*p* < 0.05), whereas the levels of SOD and GSH in the ZBSO and indomethacin treatment groups significantly increased (*p* < 0.05).

### 3.3. ZBSO Suppresses the Release of Proinflammatory Cytokines in LPS-Stimulated BEAS-2B Cells

**Figure 3:**
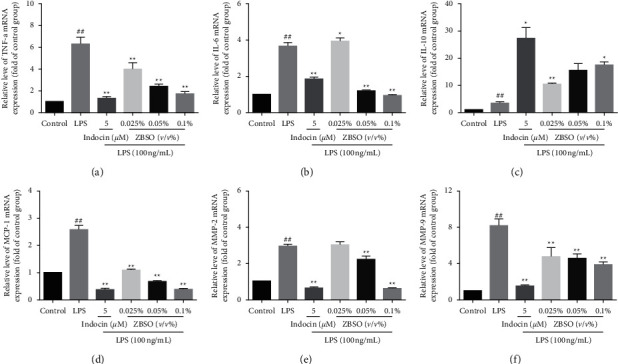
ZBSO inhibited the mRNA expression of LPS-stimulated proinflammatory factors. BEAS-2B cells were pretreated with ZBSO (0.025%, 0.05%, or 0.1% *v/v*) for 4 h and then treated with LPS (100 ng/mL) for 24 h. The results showed that ZBSO significantly inhibited LPS induction of TNF-*α* (a), IL-6 (b), IL-10 (c), MCP-1 (d), MMP-2 (e), and MMP-9 (f) expression. The experiment was repeated three times. The results are expressed as the mean ± SD; ^#^*p* < 0.05 and ^##^*p* < 0.01 versus the control group; ^*∗*^*p* < 0.05 and ^*∗∗*^*p* < 0.01 versus the LPS group.

The effects of ZBSO on the LPS-induced expression of TNF-*α*, IL-6, IL-10, and MCP-1 were investigated by RT-qPCR. As shown in Figure 3, the expression of TNF-*α*, IL-6, and MCP-1 genes in cells significantly increased after LPS stimulation (*p* < 0.05). IL-10 inhibits the inflammatory response by preventing the production of cytokines and chemokines at the transcriptional level and regulating mRNA degradation to regulate posttranscriptional expression [29]. Treatment with increasing ZBSO concentrations resulted in a significant and concentration-dependent increase in IL-10 expression levels (*p* < 0.01), with 0.1% ZBSO treatment showing the maximal effect.

### 3.4. ZBSO Protects BEAS-2B Cells against LPS-Induced Expression of COX-2 and PGE2

**Figure 4:**
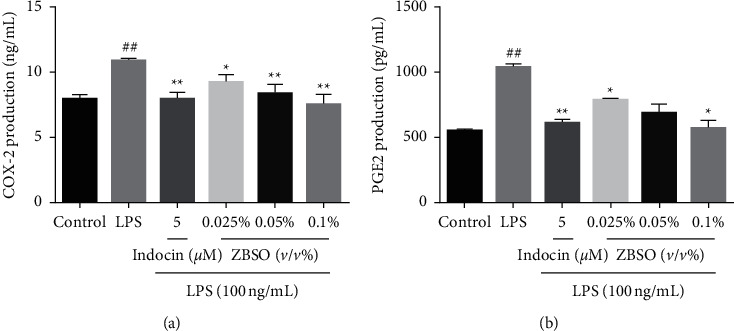
The effects of ZBSO on COX-2 and PGE2 expression in the supernatant of BEAS-2B cells. The secretion of (a) COX-2 and (b) PGE2. Data are expressed as the mean ± SD (*n* = 3); ^#^*p* < 0.05 and ^##^*p* < 0.01 versus the control group; ^*∗*^*p* < 0.05 and ^*∗∗*^*p* < 0.01 versus the LPS group.

COX-2 is the rate-limiting enzyme that catalyzes the production of prostaglandins from arachidonic acid, accelerating the production of PGE2. COX-2 expression is rapidly upregulated following stimulation, which can induce acute inflammation [30]. The effects of ZBSO on COX-2 and PGE2 induction following LPS treatment were measured by ELISA. As shown in Figures 4(a) and 4(b), ZBSO significantly reduced the protein expression of COX-2 in a dose-dependent manner. Consistently, exposure to LPS alone significantly increased the production of PGE2, and treatment with various concentrations of ZBSO significantly reduced PGE2 expression.

### 3.5. ZBSO Inhibits the Expression of MMP-2 and MMP-9 Induced by LPS

The overexpression of MMP-2 and MMP-9 is positively correlated with certain inflammatory mediators released during the initial stages of inflammation. The degradation of the extracellular matrix and basement membrane proteins can further aggravate the infiltration range of inflammatory cells, aggravating the inflammatory response [31]. Next, we evaluated the effects of ZBSO on the expression of MMP-2 and MMP-9. The results of RT-qPCR analysis (Figures 3(e) and 3(f)) showed that exposure to LPS alone increased the mRNA levels of MMP-2 and MMP-9 by 3.0- and 8.19-fold, respectively, relative to untreated cells. However, the levels of MMP-2 mRNA in the groups treated with 0.025%, 0.05%, and 0.1% (*v/v*) ZBSO were only 3.0-, 2.2-, and 0.63-fold those of untreated cells, respectively, whereas the levels of MMP-9 mRNA were reduced to 4.7-, 4.6-, and 3.8-fold those of untreated cells. The effects observed in the high-dose ZBSO treatment group were relatively better than those observed following treatment with the positive control.

### 3.6. Molecular Docking

**Figure 5:**
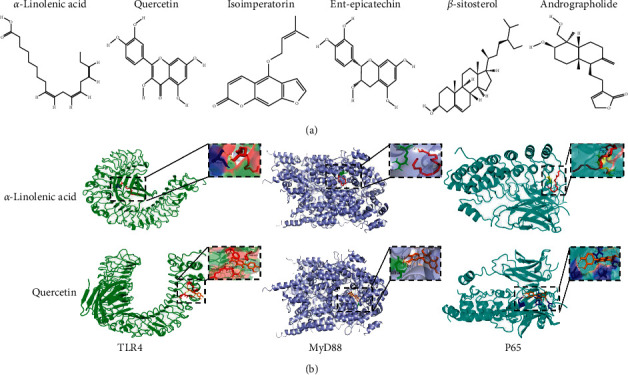
Docking results for the active components in ZBSO with key protein molecules in the TLR4/MyD88/NF-*κ*B signaling pathway. (a) The structures of active components in ZBSO were obtained by screening. (b) *α*-Linolenic acid, quercetin, and TLR4, MyD88, or NF-*κ*B p65 proteins in molecular docking mode.

Molecular docking experiments were conducted to further explore the anti-COPD mechanisms of ZBSO treatment. By screening the ingredients in ZBSO, combined with a review of the literature, seven active ingredients were identified, including *α*-linolenic acid [32], quercetin, isoimperatorin, eucalyptol, ent-epicatechin, *β*-sitosterol, and andrographolide. The basic information for these components is shown in Table 3. Molecular docking was performed to examine interactions between these components and key proteins in the TLR4/MyD88/NF-*κ*B signaling pathway, as shown in Figure 5. If the binding energy is less than 0, the ligand and the receptor can bind freely, with lower binding energy indicating a greater affinity between the receptor and the ligand and a higher interaction probability between the two components. As shown in Table 4, the binding energies determined for *α*-linolenic acid, quercetin, isoimperatorin, eucalyptol, ent-epicatechin, *β*-sitosterol, andrographolide, and indomethacin with anti-COPD target proteins were all lower than 0, indicating that the main active ingredients in ZBSO present good binding activity with TLR4, MyD88, and p65 receptor proteins. The binding effects were better than or equal to those observed for indomethacin.

### 3.7. ZBSO Suppresses LPS-Induced NF-*κ*B Nuclear Translocation in BEAS-2B Cells

**Figure 6:**
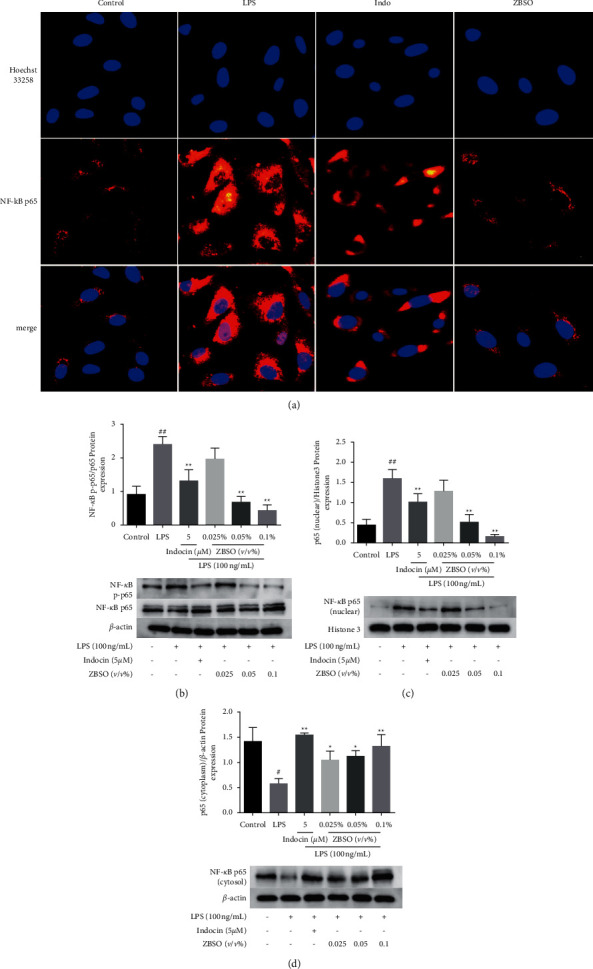
ZBSO inhibition of LPS-induced p65 nuclear translocation. (a) Typical immunofluorescence images of NF-*κ*B p65 (red) and Hoechst 33258 (blue) induced by LPS (20 × magnification). Western blot analysis of NF-*κ*B p65 total protein, NF-*κ*B p65 phosphorylation (b), and NF-*κ*B p65 in the nucleus (c) and cytoplasm (d). The values are expressed as the mean ± SD (*n* = 3) of three independent experiments. ^#^*p* < 0.05 and ^##^*p* < 0.01 versus the control group; ^*∗*^*p* < 0.05 and ^*∗∗*^*p* < 0.01 versus the LPS group.

NF-*κ*B is a nucleoprotein factor that regulates the expression of a wide range of genes and serves in a pivotal role for LPS-induced inflammatory processes [33]. LPS induces the translocation of NF-*κ*B/p65 from the cytoplasm to the nucleus, and the nuclear translocation of NF-*κ*B is associated with the release of large quantities of inflammatory mediators, such as TNF-*α*, IL-6, IL-10, NO, COX-2, and PGE2. The expression of p65 and its phosphorylated proteins in the nucleus and cytoplasm were analyzed by western blot. The nuclear translocation of p65 was also observed by immunofluorescence. As presented in Figure 6(b), the phosphorylated p65 contents of BEAS-2B cells significantly increased after LPS stimulation, whereas excessive phosphorylation was significantly inhibited by ZBSO treatment in a concentration-dependent manner. As shown in Figures 6(c) and 6(d), LPS stimulation caused p65 to shift to the nucleus and ZBSO treatment effectively blocked the nuclear accumulation of p65 induced by LPS. In addition, immunofluorescence analysis showed that ZBSO inhibited NF-*κ*B/p65 transport into the nucleus (Figure 6(a)).

### 3.8. ZBSO Reduces LPS-Induced Expression of TLR4 and MyD88

**Figure 7:**
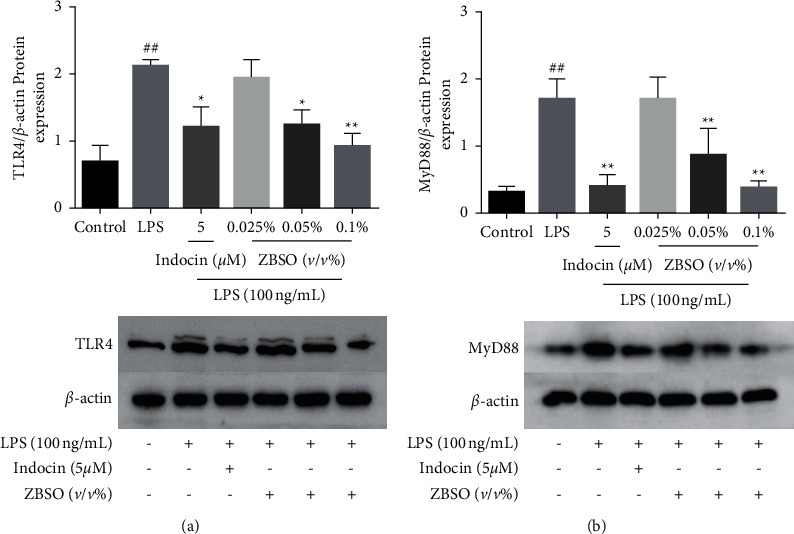
Effects of different doses of ZBSO (0.025%, 0.05%, or 0.1% *v/v*) and indomethacin on TLR4 and MyD88 protein levels in LPS-induced BEAS-2B cells. Western blot analysis of (a) TLR4 and (b) MyD88 expression levels. The values are expressed as the mean ± SD (*n* = 3) of three independent experiments. ^#^*p* < 0.05 and ^##^*p* < 0.01 versus the control group; ^*∗*^*p* < 0.05 and ^*∗∗*^*p* < 0.01 versus the LPS group.

As a component of the upstream pathway for NF-*κ*B, TLR4 binds MyD88. The MyD88-dependent TLR4 pathway promotes the production of inflammatory factors, activating the NF-*κ*B signaling pathway and leading to the expression of a variety of inflammatory factors [34]. Western blot analysis was performed to evaluate the expression of TLR4 and MyD88. As shown in Figure 7, compared with the normal control group, TLR4 and MyD88 protein expression in BEAS-2B cells increased significantly after LPS stimulation (*p* < 0.05). At low concentrations, ZBSO treatment (0.025%, *v/v*) did not significantly decrease TLR4 and MyD88 protein expression levels, whereas ZBSO treatments at 0.05% and 0.1% resulted in the significantly reduced expression of these two proteins (*p* < 0.01 − 0.05), to levels similar to those observed following treatment by the positive control drug.

## 4. Discussion

COPD is a global public health issue [35]. Current treatments include surgery and certain medications, including bronchodilators, anti-inflammatory agents, antioxidants, protease inhibitors, and antibiotics. However, the COPD prognosis is poor and no specific cure for COPD currently exists [36]. Exposure to LPS causes persistent inflammation and oxidative stress [5, 37]. In this study, to further explore the effects of ZBSO on COPD and its underlying mechanisms, LPS-treated lung epithelial cells were used to simulate the COPD microenvironment *in vitro*.

Natural medicines or traditional Chinese medicines exhibit unique and diverse chemical and biological activities, representing an important resource for new lead compounds, especially for the treatment of critical diseases. *Zanthoxylum bungeanum* belongs to the rue family and is widely used as both a spice and traditional Chinese medicine due to its unique flavor and medicinal properties. ZBSO is rich in unsaturated fatty acids [38], particularly *α*-linolenic acid, which has been reported to have anti-inflammatory and antithrombotic properties and is beneficial for the treatment of asthma and thrombosis [39]. Wang et al. [40] obtained obtucarbamate A, ent-epicatechin, quercetin, 9,19-cyclolanost-24-en-3-one, suberic acid, stearic acid, *β*-sitosterol, daucosterol, isoimperatorin, and isopimpinellin from ZBSO by extraction and isolation. The results of these component analyses are useful for understanding the biological activities of ZBSO. In recent years, molecular docking has become an important technology in the field of computer-aided drug research. Molecular docking is a drug design method that directly analyzes the characteristics of the receptor and the interaction between the receptor and the drug molecule and can be used to predict binding patterns and affinities [41]. The known components were screened through the TCMSP database, and the active compounds were obtained in combination with a review of the existing literature. The molecular docking technology was used to score the docking of the identified active compounds with the target protein, and the binding energies were all stronger than −5 kJ/mol, indicating that the active components may have high affinities for these proteins. Therefore, the components in ZBSO may act on the TLR4/MyD88/NF-*κ*B signaling pathway. However, because of the limitations of molecular docking, the flexibility of the protein and changes in the acid-base balance of the environment are generally not considered. Therefore, we used western blot analysis to verify the biological results.

TNF-*α*, IL-6, and MCP-1 are proinflammatory cytokines, which are closely related to pulmonary inflammation caused by COPD [42]. When stimulated, COX-2 expression is rapidly upregulated, which can induce acute inflammatory response in humans and animals. MMP-2 and MMP-9 are considered to be important collagenases in inflammatory diseases, and the synthesis and secretion of MMP-2 and MMP-9 are highly related to TNF-*α* [43]. IL-10 expression can reduce lung neutrophil infiltration and inhibit the expression of TNF-*α*, IL-6, and MCP-1. NO is a bioactive inflammatory inducer in the body, directly triggering the occurrence of the inflammatory process [44]. COX-2 is an inducible enzyme that is expressed at low levels in normal tissues, whereas expression increases when cells are stimulated by inflammation. PGE2 is a bioactive lipid that induces a range of biomolecules associated with inflammation. When the expression of COX-2 increases, PGE2 is released as the major COX product secreted by lung epithelial cells [45]. The results of this study demonstrated that ZBSO significantly inhibited the expression of inflammatory mediators in LPS-induced BEAS-2B cells, consistent with the results reported by Wang et al. for ZBSO in ovalbumin-induced pneumonia in an asthmatic mouse model [22]. TLR4 mediates the pathogen-induced activation of NF-*κ*B in endothelial cells through the homologous structure of intracellular IL-1 receptor, which subsequently initiates the downstream MyD88 signaling pathway, resulting in the secretion of immune-inflammatory cytokines and the development of COPD [46]. LPS could activate the TLR4/MyD88/NF-*κ*B signaling pathway in BEAS-2B cells and promote the expression of TLR4, MyD88, p65, and p-p65. In this study, immunofluorescence and western blot analyses were used to observe the expression levels of TLR4, MyD88, and p65, which were enhanced after LPS stimulation in BEAS-2B cells compared with those in the normal control group. ZBSO treatment significantly suppressed the expression levels of TLR4, MyD88, and p65 and prevented the nuclear translocation of p65. In addition, ZBSO treatment significantly blocked LPS-mediated oxidative stress and altered the levels of the oxidative stress markers ROS, MDA, GSH, and SOD.

## 5. Conclusions

In summary, the present work well illuminated that ZBSO exerted the anti-COPD effects, at least in part, by downregulating TLR4/MyD88/NF-*κ*B-mediated inflammatory responses; we believe that ZBSO has a beneficial effect on the treatment of COPD and may be a potential drug for the treatment of inflammatory diseases. Nevertheless, the composition of ZBSO is complex and diverse; meanwhile, the pathogenesis of COPD involves various signaling molecules. Therefore, in our future endeavor, more in-depth investigations on the anti-COPD effect of ZBSO, including seeking main active ingredients of ZBSO and their corresponding targeting molecules, would be performed. On the other hand, *in vivo* animal models would be employed in the future studies to depict a more comprehensive picture. These enlightenments in the future should broaden our understanding of the anti-COPD mechanism and potential of ZBSO.

## Figures and Tables

**Table 1:** Primers used in this study.

Gene primer name	Primer sequence (5′-3′)
TNF-*α* F	CGAGTGACAAGCCTGTAGCC
TNF-*α* R	TGAAGAGGACCTGGGAGTAGAT
IL-6 F	GGAGACTTGCCTGGTGAA
IL-6 R	GCATTTGTGGTTGGGTCA
IL-10 F	GTCCTCCTGACTGGGGTGAG
IL-10 R	GCCTTGATGTCTGGGTCTTG
MCP-1 F	CCTTCTGTGCCTGCTGCTCA
MCP-1 R	CACTTGCTGCTGGTGATTCTTC
MMP-2 F	TGGATGATGCCTTTGCTCG
MMP-2 R	GAGTCTCCCCCAACACCAGT
MMP-9 F	CAACATCACCTATTGGATCC
MMP-9 R	GGGTGTAGAGTCTCTCGCT
GAPDH F	CTGACTTCAACAGCGACACC
GAPDH R	TGCTGTAGCCAAATTCGTTGT

**Table 2:** The relevant targets of TLR4/MYD88/NF-*κ*B pathway.

Targets	PDB number
TLR4	2z64
MyD88	3mop
P65	1K3Z

**Table 3:** Basic information of some compounds in ZBSO.

MOL id	Chemical compound	OB (%)	DL (%)
MOL000432	*α*-Linolenic acid	45.01	0.15
MOL000098	Quercetin	46.43	0.28
MOL001942	Isoimperatorin	45.46	0.23
MOL000073	Ent-epicatechin	48.96	0.24
MOL000359	Beta-sitosterol	36.91	0.75
MOL002395	Andrographolide	56.3	0.31

**Table 4:** Molecular docking results of major active components of ZBSO and indomethacin with related targets of TLR4/MyD88/NF-*κ*B pathway.

Ligands	Binding energy (kJ/mol^−1^)		
	TLR4	MyD88	P65
*α*-Linolenic acid	−22.468	−14.309	−18.702
Quercetin	−27.447	−17.824	−26.903
Isoimperatorin	−26.317	−26.652	−25.313
Ent-epicatechin	−25.732	−19.372	−25.899
Beta-sitosterol	−16.903	−17.573	−23.389
Andrographolide	−27.747	−25.313	−25.230
Indomethacin	−25.713	−24.054	−28.451

## Data Availability

There are no basic data to support our results.

## Abbreviations

ZBSO: *Zanthoxylum bungeanum* seed oil
COPD: Chronic obstructive pulmonary disease
LPS: Lipopolysaccharide
TLR4: Toll-like receptor 4
MyD88: Myeloid differentiation factor 88
ZBM: *Zanthoxylum bungeanum* Maxim.
DMEM: Dulbecco's modified Eagle's medium
DCFH-DA: Dichlorodihydrofluorescein diacetate
OD: Optical density
PGE2: Prostaglandin E2
SOD: Superoxide dismutase
GSH: Glutathione peroxidase
MDA: Malondialdehyde
OB: Oral bioavailability
DL: Drug-likeness.

## Supplementary Materials

Supplementary Materials Figures S1–S4: *Zanthoxylum bungeanum* Maxim., *Zanthoxylum bungeanum* seed, *Zanthoxylum bungeanum* seed sample specimen, and *Zanthoxylum bungeanum* seed oil used in this study.
