# Supplementary material for: Zanthoxylum bungeanum Seed Oil Attenuates LPS-Induced BEAS-2B Cell Activation and Inflammation by Inhibiting the TLR4/MyD88/NF-κB Signaling Pathway
Source: Evid Based Complement Alternat Med. 2021 Sep 24;2021:2073296. doi: 10.1155/2021/2073296 (PMC8486531; doi:10.1155/2021/2073296)
Supplement: Supplementary Materials — Figures S1–S4: Zanthoxylum bungeanum Maxim., Zanthoxylum bungeanum seed, Zanthoxylum bungeanum seed sample specimen, and Zanthoxylum bungeanum seed oil used in this study. [file 2073296.f1.docx]

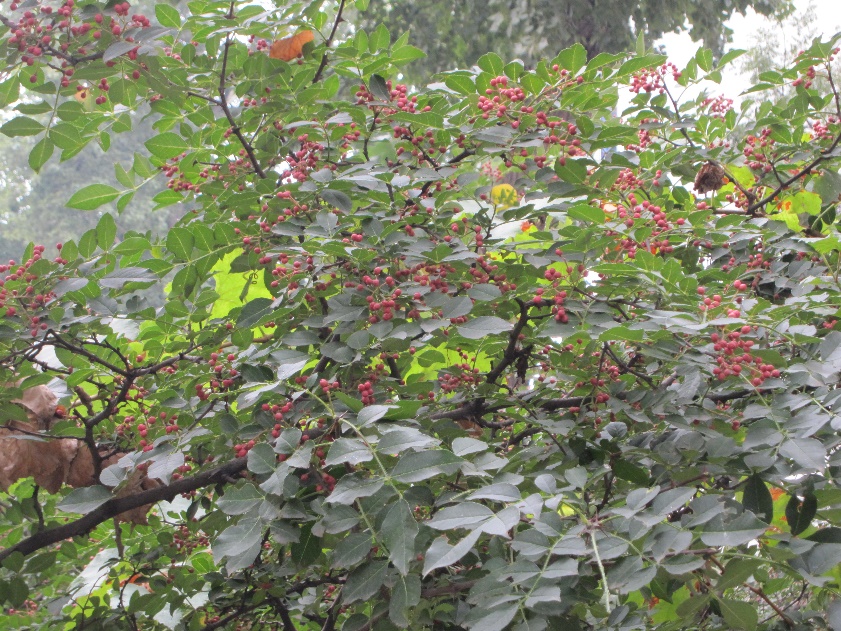


Figure 1 Z*anthoxylum bungeanum* Maxim.


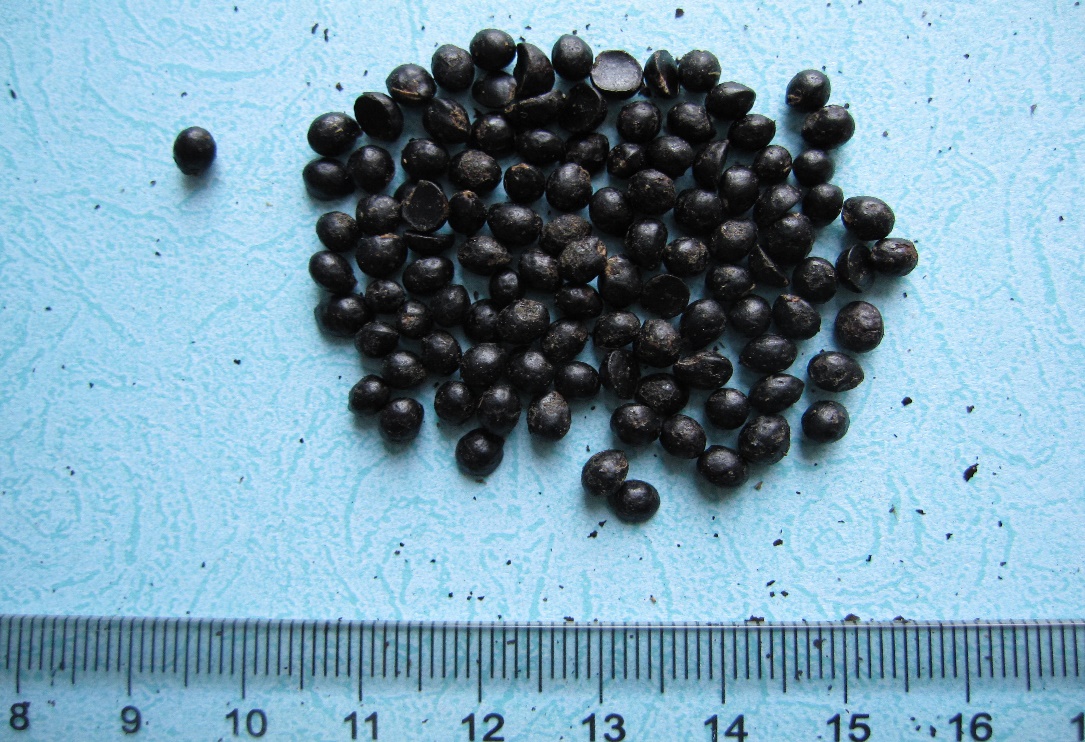


Figure 2 *Zanthoxylum bungeanum* seed


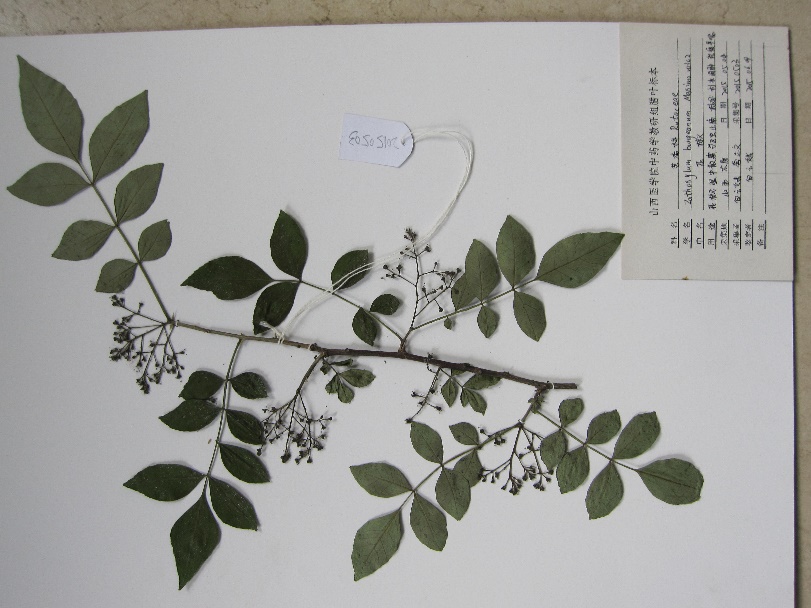


Figure 3 *Zanthoxylum bungeanum* seed sample specimen


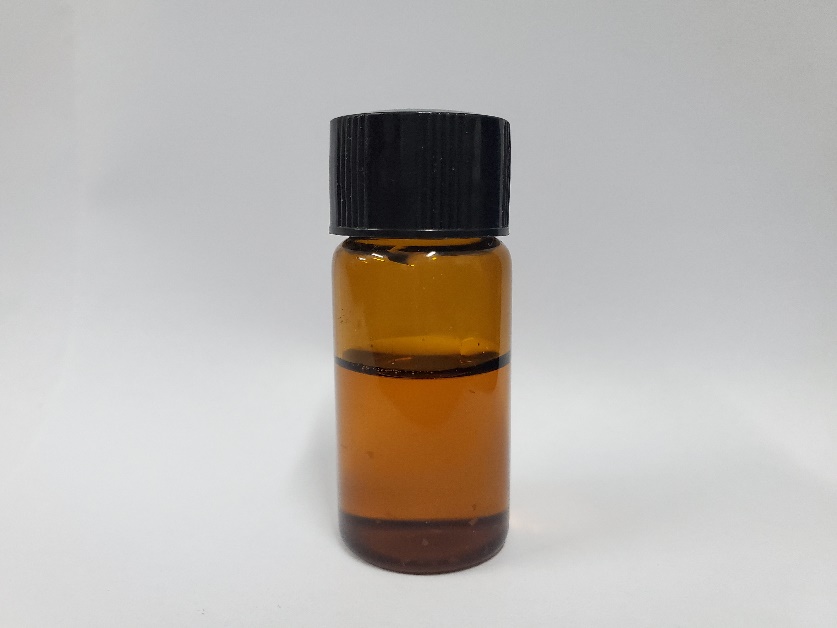


Figure 4 *Zanthoxylum bungeanum* seed oil
